# Robot-assisted investigation of sensorimotor control in Parkinson's disease

**DOI:** 10.1038/s41598-023-31299-z

**Published:** 2023-03-23

**Authors:** Yokhesh K. Tamilselvam, Mandar Jog, Rajni V. Patel

**Affiliations:** 1 Canadian Surgical Technologies and Advanced Robotics (CSTAR), University of Western Ontario (UWO), London, ON N6A 5B9 Canada; 2 Department of Electrical and Computer Engineering, University of Western Ontario (UWO), London, ON N6A 5B9 Canada; 3 Department of Clinical Neurological Sciences, UWO, and the London Movement Disorders Centre, London, ON Canada; 4 Department of Clinical Neurological Sciences, UWO, London, ON Canada; 5 Department of Surgery, UWO, London, ON Canada

**Keywords:** Parkinson's disease, Sensorimotor processing, Biomedical engineering

## Abstract

Sensorimotor control (SMC) is a complex function that involves sensory, cognitive, and motor systems working together to plan, update and execute voluntary movements. Any abnormality in these systems could lead to deficits in SMC, which would negatively impact an individual's ability to execute goal-directed motions. Recent studies have shown that patients diagnosed with Parkinson's disease (PD) have dysfunctions in sensory, motor, and cognitive systems, which could give rise to SMC deficits. However, SMC deficits in PD and how they affect a patient's upper-limb movements have not been well understood. The objective of the study was to investigate SMC deficits in PD and how they affect the planning and correction of upper-limb motions. This was accomplished using a robotic manipulandum equipped with a virtual-reality system. Twenty age-matched healthy controls and fifty-six PD patients (before and after medication) completed an obstacle avoidance task under dynamic conditions (target and obstacles in moving or stationary form, with and without mechanical perturbations). Kinematic information from the robot was used to extract eighteen features that evaluated the SMC functions of the participants. The findings show that the PD patients before medication were 32% slower, reached 16% fewer targets, hit 41% more obstacles, and were 26% less efficient than the control participants, and the difference in these features was statistically significant under dynamic conditions. In addition to the motor deficits, the PD patients also showed deficits in handling high cognitive loads and interpreting sensory cues. Further, the PD patients after medication exhibited worse sensory and cognitive performance than before medication under complex testing conditions. The PD patients also showed deficits in following the computational models leading to poor motor planning.

## Introduction

Humans physically interact with the world around them through a multitude of motor behaviors including goal-directed movements (- a set of motor actions performed in a specific sequence with an intent to achieve a desired outcome or goal)^1,2^. Sensorimotor control^3^, which is the ability to interpret the acquired multi-modal sensory input and appropriately plan, correct, and generate motor commands to achieve the desired outcome(s), is essential in appropriately performing goal-directed movements. Consequently, the optimal functioning of SMC requires multiple systems (motor, sensory and cognitive) working together in a closed loop. While motor systems play a big part in movement execution, planning and correcting/updating a planned strategy can only be attained through two important additional contributors: (i) the sensory system, which helps in perceiving our body and the world around us; and (ii) the cognitive system, which aids in a series of decision-making processes such as planning or correcting the motor control strategies based on our perception^4,5^. Therefore, the two SMC functions, namely movement planning (ability to develop a motor plan) and online error correction (ability to rapidly update the developed motor plan to adapt to changes in the environment), are extremely important in performing even the simplest of motor tasks. Any impairments in the motor, sensory or cognitive system may adversely affect these SMC functions, which could lead to a cascade of deficits affecting goal-directed movements.

Parkinson's disease (PD) is a progressive neurodegenerative disorder resulting from the degeneration of dopaminergic neurons in the Basal Ganglia (BG). Pathology of PD is extremely complex, with cardinal symptoms that include rigidity, tremor, bradykinesia, and postural instability^6^. Recent studies have hypothesized dysfunctions in motor^7^, cognitive^8,9^ and sensory systems^10–13^ that may negatively impact SMC functionalities such as movement planning and error correction. These SMC deficits could severely affect the patient's ability to perform any voluntary movements. Studies that discuss SMC deficits either do not use an objective method to quantify the impairments^14^ or do not perform an in-depth analysis to understand how the SMC functionalities in PD patients differ from those in healthy subjects^15^. Characterizing these SMC deficits and understanding the underlying mechanisms that affect SMC can open new doors for rehabilitation strategies in PD. Since the efficacy of any rehabilitative process depends on how accurately it targets the deficits^4^, there is currently a need to characterize and better understand the basis behind the SMC deficits experienced in PD.

Another important reason for studying SMC deficits in PD is to understand the effect of medication on these deficits. While it is well known that medications such as levodopa mitigate motor symptoms, it is unclear if a decrease in rigidity and bradykinesia from dopaminergic medication also mitigates SMC deficits. This is because SMC encompasses a broader and more complex network that involves multiple systems coordinating with each other to plan and update the motor strategy for performing task-specific motions. Understanding the effect of medication on SMC functions in PD patients could explain if this medication alters the functioning of multiple systems, thereby mitigating or worsening the SMC deficits. Therefore, it is also essential to investigate the effect of medications on these SMC deficits and how it affects the patient's ability to perform goal-directed movements.

This study aims to objectively quantify impairments in SMC functions such as movement planning and online error correction in PD patients. Since optimal movement planning and error correction depend on the proper functioning of the motor, cognitive and sensory systems^5^, testing tasks used in this study focused on the evaluation of deficits pertaining to the functioning of these systems. Upper-limb movements of PD patients and age-matched healthy participants were collected when performing an obstacle avoidance task using a robotic manipulandum. The features extracted were compared between the groups to understand the motor, sensory and cognitive deficits due to PD and the effect of medication. While a direct comparison between groups allows for quantification of the performance, the comparison of the features from the perspective of computational models may help in understanding how PD affects the planning or correction of a movement. So far, there has not been any study that has analyzed SMC deficits in PD patients through the prism of generic and task-specific computational models.

## Methods

### Subjects

Fifty-six participants diagnosed with PD and twenty age-matched healthy subjects were recruited for this study. The Office of Human Research Ethics in Western University's Research Ethics Board approved this study (protocol number: 115770, 108,52). The study has been conducted in accordance with the ethical standards laid down in the 1964 Declaration of Helsinki. All subjects provided their informed consent before the study. All patients were recruited through the Movement Disorders Clinic at the London Health Sciences Centre in London, Ontario, Canada. Inclusion criteria for patients were diagnosis of PD, no injury-limiting upper-limb movements, and normal or corrected-to-normal vision. The severity of the disease was assessed in each patient using the United Parkinson’s Disease Rating Scale-III (UPDRS-III) before and one hour after the medication. Furthermore, the cognitive status of the patients was also assessed using Montreal Cognitive Assessment (MoCA), which indicated that the mean MoCA score across all the PD patients was 26. No dyskinesia was observed in PD patients after medication. Freezing of gait was observed in a few patients (UPDRS-3.11 in the OFF state: mean score = 0.22, range = 3; UPDRS-3.11 in the ON state: mean score = 0.02, range = 1), but it did not affect the upper-limb performance of any patient. The control subjects were recruited, excluding participants diagnosed with a condition or injury affecting their movement, vision, or brain functions. Demographic and clinical information pertaining to individual PD patients are provided in the supplementary section under Table S3.

### Experimental setup and study design

**Figure 1:**
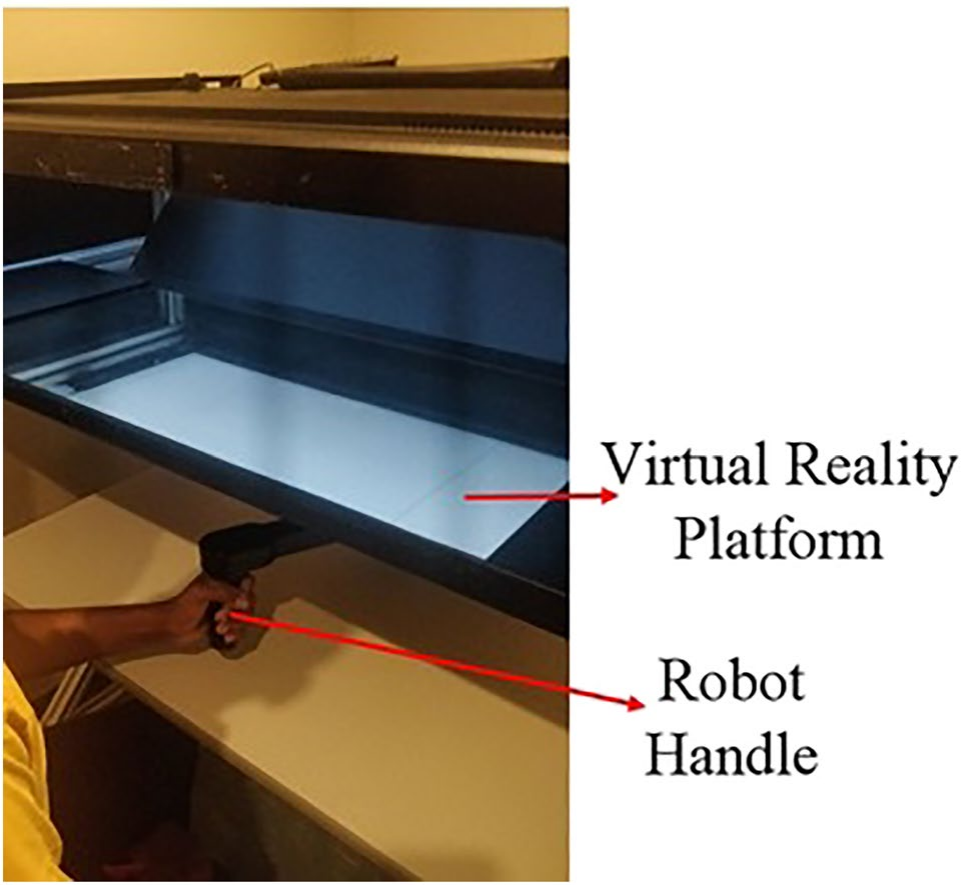
A closer look at the KINARM end point robot with a subject.

The movement planning and online error correction in the subjects were assessed using a two-joint robotic manipulandum (KINARM endpoint robot)^16^ supplemented with a virtual reality display. Figure 1 shows the KINARM endpoint robot. This robot allows manipulation of the participant's arm in two dimensions along a horizontal plane. Real-time visual feedback of the subject's fingertip position along with the virtual objects (targets and obstacles) related to the task was provided to the participants using the virtual reality system. Furthermore, a black screen was placed between the virtual reality display and the participant’s arm to prevent the participants from seeing their own arm when performing the robotic assessments. The participants sat in an upright position and were asked to hold the upper end of the robot handle, which was in parallel with the subject's waist level.

**Figure 2:**
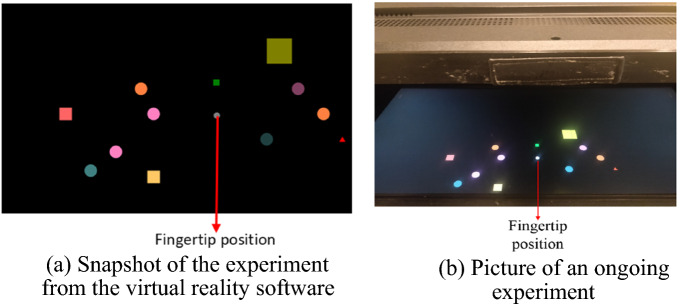
Obstacle avoidance task; the targets are square-shaped, and the obstacles are in the shape of circles and triangles.

In the literature^17,18^, it has been shown that performing a dynamic obstacle avoidance task requires proper functioning of motor, sensory and cognitive resources and, therefore, can be used to objectively characterize deficits in goal-directed movements. A custom obstacle avoidance task was built using Simulink^19^ for testing the participants. The participants were asked to reach the targets while avoiding the obstacles. The targets were square-shaped, while the obstacles were in the shape of circles or triangles. The size and color of each target and obstacle varied for each trial. Participants performed a total of 40 trials with each arm. The PD patients performed the assessment before and one hour after taking their medication. Each trial included four targets and eight obstacles. Therefore, in total, the task consisted of 160 targets and 320 obstacles. On average, the task took about 6–8 min per arm. In addition to visual information, participants were provided with a haptic (vibrotactile signal in the handle) and an auditory sensory cue (beeping sound) when they reached 2–2.5 cm from an obstacle to determine if they could interpret the sensory information and appropriately avoid the obstacle. The task was divided into four sub-tasks (Levels, L) with increasing levels of difficulty. Each sub-task included stationary or moving targets and obstacles (Table 1). The target and obstacle could be moving or stationary in each level. In levels where the targets or obstacles were moving, the movement of these virtual objects was in a circular trajectory, with the radius and center of the trajectory being different from one object to another. The specifications for each level are explained in Table 1. The width and speed of the targets and the obstacles range from 1 to 6 cm and 1.8 to 22.8 cm/s, respectively, and were varied randomly. Further, a mechanical perturbation was also applied to the handle. The first level had no perturbation, while the robot applied one, two, or three perturbations in random directions during L-2, L-3, and L-4, respectively, with each perturbation, separated by a few seconds from the previous. In levels (L-3 and L-4) with more than one perturbation, the time intervals between subsequent perturbations were varied to ensure that the participants do not get used to the time interval between perturbations and attempt to generate a corrective movement even before a perturbation is applied. The time interval between the perturbations was between 2.5 to 5 s. The force applied to generate a perturbation varied for each level and is indicated in Table 1. As the level increased, the force applied to generate a perturbation also increased. The supplementary material includes a video showing the task design of each level. Figure 2 shows an image of the obstacle avoidance task, including the fingertip position and the virtual objects that the participants can view through the display.

**Table 1:** Design of each level in obstacle avoidance task.

Level	Target	Obstacle	Perturbations
Level-1 (L-1)	Stationary	Stationary	None
Level-2 (L-2)	Moving	Stationary	One (2–2.8 N)
Level-3 (L-3)	Stationary	Moving	Two (3–4.2 N)
Level-4 (L-4)	Moving	Moving	Three (4–5.6 N)

The testing conditions for each level vary in terms of the stationary or moving targets and obstacles and the applied perturbations; the minimum and maximum net force applied to generate perturbations are provided in the last column.

### Feature extraction

The KINARM endpoint robot collected kinematic and force data at a sampling rate of 1000 Hz. Prior to the feature extraction process, the data was filtered using a dual-pass digital Butterworth filter at a cut-off frequency of 10 Hz^20^. A total of 18 features, which are indicated in Table 2, were extracted using MATLAB to objectively determine motor, sensory, and cognitive deficits. Based on the literature^21,22^, features including speed, movement area, and time were used as indicators of motor performance. There are studies^23^ that suggest impairments in sensory functions due to PD. Therefore, features such as obstacle hit-to-warn ratio and corrective time that could help characterize these sensory impairments were extracted as an indicator for sensory performance^24,25^. Studies^26–28^ have explained cognitive processes in goal-directed movement as an executive function to develop a strategy to efficiently and accurately accomplish the goal (reaching the target and avoiding obstacles). Therefore, features such as target reach, obstacle hit, error, and efficiency were used to evaluate the subject's cognitive ability. While it must be noted that these features may not be a pure measure of the respective domains (motor, sensory and cognitive) that they were assigned to assess, each feature was assigned to determine the performance of a specific domain based on which domain influences that feature the most. For instance, components of testing that were not directly related to the production of motion but yet influenced the overall task performance through planning and correction of the movement were grouped as cognitive features. The right- and left-hand performances for each feature were averaged together^15^.

**Table 2:** Features extracted during the study.

Features	Definitions	Purpose
Mean speed	Mean velocity throughout the entire task	Motor deficits
Peak speed	Maximum velocity throughout the entire task	Motor deficits
Time to reach maximum speed	Time taken to reach the peak velocity	Motor deficits
Movement area	Area covered throughout the task using convex hull	Motor deficits
Reaction time	Time required to reach 10% of the total distance	Motor deficits
Speed peaks	Number of maxima in hand speed	Motor deficits
Movement time	Time taken from the movement onset to the end	Motor deficits
Obstacle hit to warn ratio	Ratio of (i) number of obstacles hit in each trial. (ii) number of warnings provided through auditory, visual, and vibrotactile sensory cues in each trial	Sensory deficits
Corrective time for perturbation	Time required to correct for any perturbation	Sensory deficits
Target reach percent	Mean percentage of targets reached	Cognitive deficits
Efficiency	Ratio of (i) distance traveled to reach a target, (ii) distance pertaining to the shortest path to reach a target	Cognitive deficits
Target order	The ideal target order was determined, taking into consideration the target and obstacle location. Target order that requires the participants to travel the least amount of distance was considered the ideal target order. R^2^ values were calculated between the ideal order and order in which subjects reach target	Cognitive deficits
Endpoint error	Distance between the fingertip and center of the target when the subject reaches and stays at the target	Cognitive deficits
Obstacle hit	Mean number of obstacles hit during the task	Cognitive deficits
Corrective movements	Number of corrective movements performed throughout the task	Cognitive deficits
Endpoint variance	Variance of distance between the fingertip position and target center	Cognitive deficits
Slope between performance and Index of difficulty (ID)	ID was calculated using Fitts's law, and the performance indicator is taken as movement time as per Fitts's law. The equation to calculate ID is provided in the supplementary material	Cognitive deficits
Error-speed ratio	Ratio between Endpoint error and Mean speed. Rate at which the endpoint error increases for every 1 cm/s increase in mean velocity	Cognitive deficits

### Computational models

**Figure 3:**
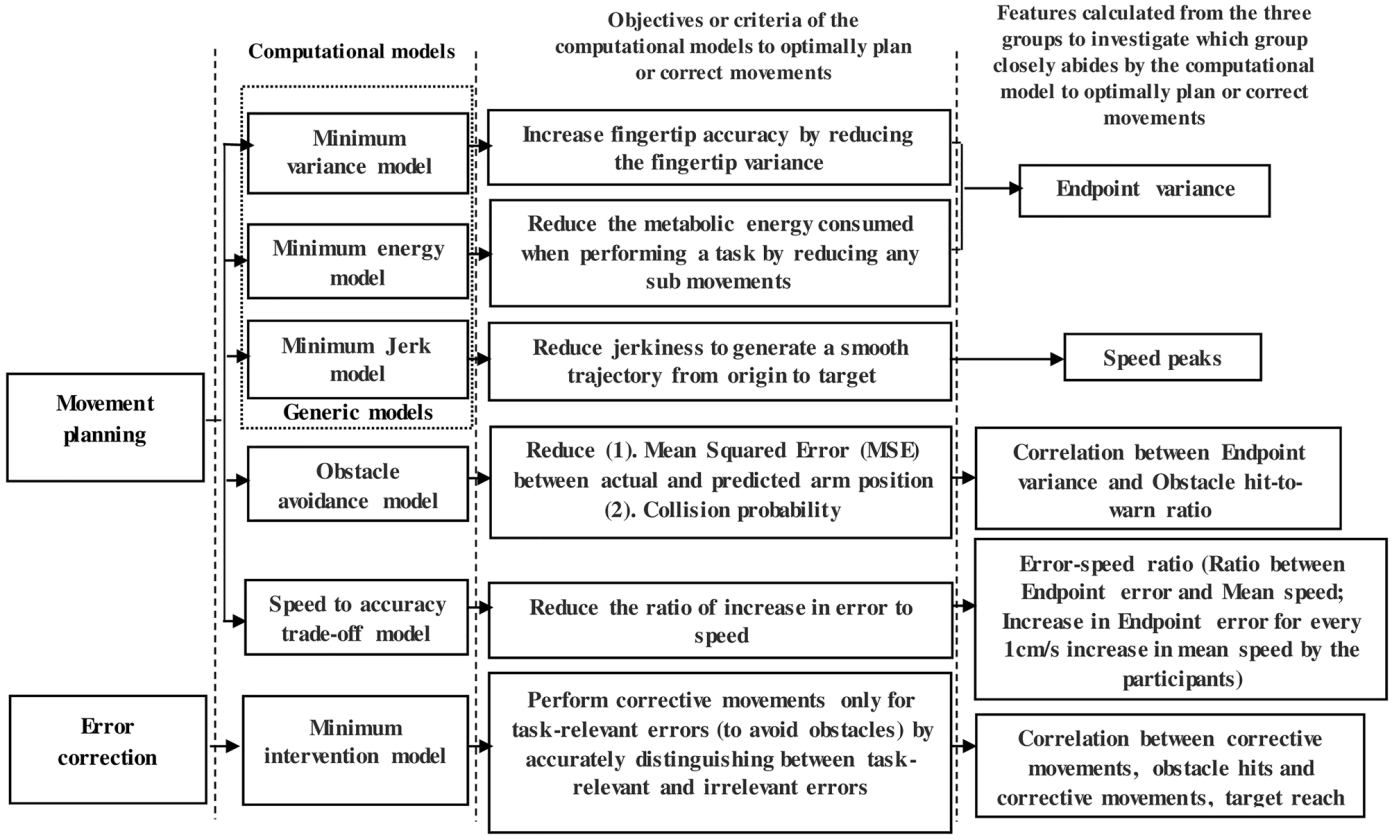
Computational models and features extracted to compare the performance of the groups. Features were extracted from the three groups and compared with each other to determine which group performs the best based on the objectives or criteria proposed by the computational model.

One of the study's objectives was to understand the causes for any performance deterioration in PD patients. While the exact functioning of the Central Nervous System (CNS) or the criteria used by our brain in optimally performing a goal-directed movement is still a topic of debate, numerous computational models have been hypothesized in earlier studies to theoretically highlight the criteria that may be used by the CNS to optimally plan and correct a movement. Certain computational models may also include a cost function equation that represents the objective or criterion proposed by the model to attain optimal performance, and this criterion varies from one model to another. For instance, in a minimum variance model^29^, the objective or criterion of the model for optimal movement is to minimize the variance of the fingertip. It is also hypothesized that the CNS may use different criteria that account for the goals of the task and the biomechanical redundancies to obtain an optimal task performance^30–32^. While kinematic data^27^ from healthy subjects were assessed from the perspective of certain computational models, no such assessments have been done before for PD patients. Comparing our results from the perspective of these models could explain how the dysfunction in PD affects the criteria that may be used by the CNS, which in turn leads to any underperformance. Furthermore, it also provides a better understanding of the basis of the SMC deficits, which would assist in targeting them through a systematic rehabilitation regime. Therefore, the extracted features from the three groups were compared with each other from the perspective of the SMC-based computational models. That is, the features that represent the objectives of the six computational models were extracted from the three groups and compared with each other to determine which group performed better from the perspective of each computational model. For instance, in a minimum variance model, the objective is to minimize the fingertip variance. Therefore, the endpoint variance, which was the fingertip variance in our task, was extracted for the three groups and compared with each other to determine which group had the least fingertip variance, thereby performing the best from the perspective of the minimum variance model. Figure 3 shows the objective of the six computational models and the features extracted to evaluate the subject's performance from the perspective of the computational models. Since the criterion used by the models represents the optimal strategy for planning and updating a movement, any deficits that were observed in subjects when compared from the perspective of the computational models can be attributed to deficits in performing the necessary executive processes required for goal-directed movement.

Looking at Fig. 3, the endpoint variance was used for comparing the performance between the groups from the perspective of the minimum energy^30,33^ and variance^29^ models, as the objective of both these models is to reduce the endpoint variance, which could, in turn, increase fingertip accuracy and reduce energy consumption. Similarly, speed peaks were used for comparing the group’s performance from the perspective of the minimum jerk model^34^, as an increase in speed peaks would lead to higher jerkiness. The cost function proposed for the obstacle avoidance model^17^ has two parts, as shown in Fig. 3 and the features that represent these two parts were extracted, and a correlation between the features that represent the two parts of the cost function was calculated to investigate how efficiently subjects were able to reduce at least one part of the cost function. The speed-to-accuracy trade-off model^29^ discusses the signal-dependent noise proportional to the movement speed that could lead to inaccuracies. The model hypothesizes that there is a trade-off between speed and accuracy, i.e., an increase in speed may be accompanied by a loss in the accuracy of the movement. Therefore, the increase in endpoint error for every cm/s increase in speed (error-speed ratio) was calculated for the three groups to determine the trade-off in accuracy when a 1 cm/s increase in speed was observed. A comparison was also made with the minimum intervention model^32,35^. The model's objective is for the subject to distinguish between task-relevant and irrelevant errors and efficiently correct only the relevant errors, such as avoiding obstacles. Therefore, correlations between obstacle hits, target reach, and corrective movements were calculated to determine the subject's ability to optimally perform corrections only for task-relevant errors.

### Statistical analyses

In this study, two different statistical analyses were performed to determine any statistically significant differences between the groups. Pairwise comparisons between Parkinson’s patients before medication (PD-OFF) and Parkinson’s patients after medication (PD-ON) groups were made using the Wilcoxon signed-rank test, while a comparison between the PD-OFF group versus control subjects and PD-ON group versus control subjects was made using the Mann–Whitney-Wilcox test^36^ with Bonferroni correction. Non-parametric tests were used as the data was not normally distributed. A *p*-value less than 0.05 was considered significant in both tests. Further, the correlation between features and the UPDRS-III score was calculated using the Spearman correlation coefficient. The correlation coefficient between features and their significance was calculated for each level separately and then combined using Fisher's Z-transformation method^37,38^ and harmonic mean method^39^.

## Results

### Demographic information and UPDRS-III

Out of the fifty-six PD patients, fifty-four patients were right-hand dominant, while two patients were left-hand dominant. Out of Twenty age-matched control subjects, nineteen were right-hand dominant, and one was left-hand dominant. Table 3 shows the patient demographics and UPDRS—III scores.

**Table 3:** Demographics and clinical data for the PD patients and Control subjects.

	PD patients	Control subjects
Number	56	20
Age (years) (mean (range))	62 (29)	58 (28)
Gender (m/f)	40/16	13/7
Years with disease (mean (range))	10 (28)	N/A
UPDRS motor sub-scale in OFF state (mean (range))	46 (67)	N/A
UPDRS motor sub-scale in ON state (mean (range))	30 (47)	N/A
MOCA (mean (range))	26 (9)	N/A

### PD-OFF vs. control subjects

This section compares the performance of the PD-OFF group with the control group to investigate SMC deficits. The impairments observed in the motor, sensory and cognitive systems leading to SMC deficits are discussed in this section. Table 4 shows the performance of the groups and the *p*-values obtained from the three statistical analyses.

**Table 4:** Extracted features for the three groups and the corresponding statistical significance.

Parameters		PD-OFF, median (range)	PD-ON, median (range)	Control, median (range)	Significance
Mean speed (cm/s)	L-1	0.125 (0.127)	0.135 (0.123)	0.166 (0.076)	*p* = 0.0016*, *p* = 0.0879, *p* = 0.0139*
	L-2	0.154 (0.206)	0.176 (0.160)	0.214 (0.076)	*p* = 0.0014*, *p* = 0.0703, *p* = 0.0096*
	L-3	0.152 (0.169)	0.167 (0.151)	0.210 (0.097)	*p* = 0.0007*,* p* = 0.0062*, *p* = 0.0065*
	L-4	0.159 (0.174)	0.178 (0.152)	0.235 (0.010)	*p* = 0.0006*,* p* = 0.010*, *p* = 0.0057*
Peak speed (cm/s)	L-1	0.563 (0.749)	0.570 (0.666)	0.887 (0.786)	*p* = 0.0004*, *p* = 0.2343, *p* = 0.0008*
	L-2	0.604 (0.815)	0.685 (0.770)	0.894 (0.624)	*p* = 0.0031*, *p* = 0.071, *p* = 0.0157*
	L-3	0.629 (0.899)	0.679 (0.528)	1 (0.748)	*p* = 0.0006*,* p* = 0.032*, *p* = 0.0003*
	L-4	0.684 (0.759)	0.776 (0.671)	1.013 (0.666)	*p* = 0.0002*,* p* = 0.044*, *p* = 0.0056*
Time to reach maximum speed (s)	L-1	5.468 (6.086)	5.168 (6.529)	4.608 (5.008)	*p* = 0.43, *p* = 0.039*, *p* = 0.9480
Movement area (cm^2^)	L-1	626.4 (753)	644.9 (237)	737.5 (233)	*p* = 0.0062*, *p* = 0.0836, *p* = 0.0050*
	L-2	753.8 (1008)	852.5 (793)	954.9 (321)	*p* = 0.0442*, *p* = 0.024*, *p* = 0.2488
	L-3	748.3 (561)	754.2 (361)	860.6 (557)	*p* = 0.0385*,* p* = 0.212, *p* = 0.0348*
	L-4	714.1 (999)	860.1 (771)	1002 (328)	*p* = 0.0048*,* p* = 0.08, *p* = 0.0938
Reaction time (s)	L-1	1.571 (1.485)	1.334 (1.216)	1.295 (0.704)	*p* = 0.010*, *p* = 0.0329*, *p* = 0.5280
Speed peaks	L-1	8 (10)	6 (8)	5 (2)	*p* = 0.0123*, *p* = 0.0654, *p* = 0.0973
	L-2	14 (6)	12 (5)	10 (5)	*p* = 0.0431*, *p* = 0.0811, *p* = 0.2142
	L-3	10 (9)	9 (6)	7 (2)	*p* = 0.0006*,* p* = 0.0035*, *p* = 0.0499*
	L-4	13 (6)	13 (5)	11 (3)	*p* = 0.1094,* p* = 0.160, *p* = 0.2723
Movement time (s)	L-1	8.969 (12.91)	7.756 (4.674)	7.484 (2.426)	*p* = 0.0027*,* p* = 0.0022*, *p* = 0.0231*
	L-2	10.66 (3.351)	10.82 (4.601)	9.001 (3.414)	*p* = 0.011*,* p* = 0.0062*, *p* = 0.3494
	L-3	8.645 (5.072)	7.964 (5.416)	7.326 (1.965)	*p* = 0.0004*,* p* = 0.0019*, *p* = 0.0856
	L-4	11.29 (3.183)	11.02 (4.083)	10.19 (2.830)	*p* = 0.0282*,* p* = 0.0401*, *p* = 0.6793
Obstacle hit-to-warn ratio	L-1	0.051 (0.300)	0.040 (0.233)	0.013 (0.144)	*p* = 0.0252*, *p* = 0.0203*, *p* = 0.0894
	L-2	0.055 (0.474)	0.043 (0.333)	0.018 (0.194)	*p* = 0.101, *p* = 0.255, *p* = 0.0642
	L-3	0.314 (0.574)	0.355 (0.300)	0.210 (0.259)	*p* = 0.0052*,* p* = 0.0935, *p* = 0.004*
	L-4	0.300 (0.446)	0.323 (0.339)	0.145 (0.257)	*p* = 0.4274,* p* = 0.7782, *p* = 0.6476
Corrective time for perturbation (s)	L-2	0.196 (0.237)	0.192 (0.273)	0.162 (0.119)	*p* = 0.040*, *p* = 0.2432, *p* = 0.0780
	L-3	0.208 (0.127)	0.226 (0.184)	0.181 (0.113)	*p* = 0.09,* p* = 0.2954, *p* = 0.0679
	L-4	0.216 (0.168)	0.238 (0.161)	0.203 (0.142)	*p* = 0.115, *p* = 0.967, *p* = 0.4208
Target reach percent (%)	L-1	0.912 (0.607)	0.975 (0.305)	0.986 (0.055)	*p* = 0.1438, *p* = 0.87, *p* = 0.09
	L-2	0.582 (0.722)	0.638 (0.710)	0.716 (0.388)	*p* = 0.0801, *p* = 0.0220*, *p* = 0.5413
	L-3	0.923 (0.361)	0.968 (0.166)	1 (0.027)	*p* = 0.0045*, *p* = 0.26, *p* = 0.0084*
	L-4	0.555 (0.611)	0.680 (0.527)	0.763 (0.222)	*p* = 0.010*, *p* = 0.0312*, *p* = 0.1211
Efficiency	L-1	0.671 (0.546)	0.685 (0.410)	0.879 (0.167)	*p* = 0.0359*, *p* = 0.936, *p* = 0.0223*
Target order	L-1	0.705 (0.577)	0.749 (0.392)	0.838 (0.278)	*p* = 0.029*, *p* = 0.935, *p* = 0.0489*
End point error (cm)	L-1	0.454 (0.387)	0.462 (0.524)	0.257 (0.112)	*p* = 0.015*, *p* = 0.0879, *p* = 0.0065*
	L-2	0.676 (0.541)	0.729 (0.672)	0.591 (0.510)	*p* = 0.0425*, *p* = 0.0703, *p* = 0.0250*
	L-3	0.421 (0.630)	0.396 (0.325)	0.272 (0.133)	*p* = 0.018*,* p* = 0.012*, *p* = 0.0387*
	L-4	0.630 (0.409)	0.640 (0.550)	0.587 (0.361)	*p* = 0.0032*,* p* = 0.159, *p* = 0.0647
Mean obstacle hit proportion per trial	L-1	17.11 (26.25)	15.66 (24.43)	11.62 (10.90)	*p* = 0.284, *p* = 0.199, *p* = 0.294
	L-2	45.68 (32.53)	46.27 (37.76)	26.76 (19.72)	*p* = 0.313, *p* = 0.193, *p* = 0.311
	L-3	85.76 (63.33)	69.31 (58.31)	53.72 (43.28)	*p* = 0.0421*,* p* = 0.701, *p* = 0.5621
	L-4	86.87 (67.89)	89.46 (71.52)	56.67 (47.71)	*p* = 0.023*,* p* = 0.253, *p* = 0.0215*
Total Corrective movements	L-1	87 (141)	69 (83)	58 (14)	*p* = 0.0068*, *p* = 0.0021*, *p* = 0.2028
	L-2	111 (224)	105 (72)	65 (46)	*p* = 0.018*, *p* = 0.0329*, *p* = 0.8276
	L-3	93 (122)	84 (98)	73 (34)	*p* = 0.0003*, *p* = 0.018*, *p* = 0.0611
	L-4	127 (104)	121 (59)	115 (73)	*p* = 0.0449*, *p* = 0.0437*, *p* = 0.3378
Endpoint variance (cm)	L-1	0.556 (2.062)	0.517 (2.517)	0.133 (0.835)	*p* = 0.044*, *p* = 0.880, *p* = 0.0139*
	L-2	1.213 (5.096)	1.706 (6.245)	1.108 (3.353)	*p* = 0.056, *p* = 0.492, *p* = 0.0361*
	L-3	0.488 (2.923)	0.445 (2.463)	0.201 (0.422)	*p* = 0.0419*, *p* = 0.730, *p* = 0.0780
	L-4	1.161 (4.451)	1.947 (5.220)	0.950 (2.475)	*p* = 0.232, *p* = 0.0071*, *p* = 0.0616
Slope between performance and ID	L-1	0.391 (0.284)	0.361 (0.249)	0.283 (0.198)	*p* = 0.217, *p* = 0.0068*, *p* = 0.683
	L-2	0.904 (0.693)	0.940 (0.560)	0.791 (0.412)	*p* = 0.026*, *p* = 0.519, *p* = 0.0011*
	L-3	0.410 (0.346)	0.391 (0.331)	0.321 (0.351)	*p* = 0.028*, *p* = 0.277*, p* = 0.0021*
	L-4	0.927 (0.540)	0.876 (0.670)	0.827 (0.790)	*p* = 0.0097*, *p* = 0.687,* p* = 0.065
Error-speed ratio	L-1	3.60 (16.2)	3.26 (9.34)	1.40 (1.57)	*p* = 0.0009*, *p* = 0.212, *p* = 0.0012*
	L-2	4.51 (11.5)	4.10 (9.90)	2.87 (4.43)	*p* = 0.0268*, *p* = 0.076, *p* = 0.3275
	L-3	2.35 (9.95)	1.98 (6.04)	1.12 (1.52)	*p* = 0.0008*, *p* = 0.032*, *p* = 0.0057*
	L-4	3.48 (10.3)	3.47 (6.96)	2.26 (3.38)	*p* = 0.125, *p* = 0.398, *p* = 0.2314

The first value in the significance column corresponds to PD-OFF vs. controls; the second value corresponds to the PD-OFF vs. PD-ON; the third value corresponds to the PD-ON vs. controls; * after the *p*-value indicates statistical significance.

Looking at the motor deficits, the PD-OFF group was much slower than the control group in all four levels. The mean and peak speed for the PD-OFF group was 32% and 41% lower compared to the control group across the four levels and was also statistically significant (see Table 4). The movement time was also 15% higher for the PD-OFF group across all four levels. Further, the movement area of the control group was statistically significantly higher (see Table 4) than the PD-OFF group. The median reaction time for the control group was 1.295 s, which was 19.2% lower than the reaction time for the PD-OFF group. In addition to this, the PD-OFF group took more time to reach maximum speed than the control group, although the difference was not statistically significant (see Table 4). There was also a positive correlation (${r}_{s}$ = 0.5700, *p* = 0.008) between reaction time and time to reach the maximum speed.

Features evaluating sensory deficits were obstacle hit-to-warn ratio and corrective time for perturbation. The PD-OFF group has an 82% more hit-to-warn ratio than the control subjects. The difference between PD-OFF and control groups in the obstacle hit-to-warn ratio was statistically significant (see Table 4) in trials where the targets were not moving. Further, the PD-OFF group also took about 13% more time to initiate a corrective movement for a perturbation. While the within-group analysis indicates that both groups took more time to correct for perturbations as the difficulty increased due to an increase in level, the PD-OFF group took 18%, 13%, and 6% more time to initiate a corrective movement in L-2, L-3, and L-4 respectively, than the control group. Therefore, as the force applied in a perturbation increase, the performance of the PD-OFF group gets better and becomes closer to the control group. The corrective time for each individual (first, second, and third) perturbation was analyzed by averaging the corrective time for individual perturbations across multiple levels. While the corrective time for the first perturbation was averaged across L-2, L-3, and L-4, the corrective time for the second perturbation was averaged across L-3 and L-4. The corrective time for the third perturbation was obtained from L-4, as only L-4 included the third perturbation. The results indicate that the difference in corrective time between PD-OFF and control groups was 2.54%, 7.12%, and 11.93% for the first, second, and third perturbations, respectively.

Moving on to features evaluating cognitive deficits, the control subjects reached more targets in all four levels than the PD-OFF group. There was a statistically significant difference (see Table 4) in the target reach percentage between the control and PD-OFF group in L-3 and L-4, while in L-1 and L-2, the PD-OFF group reached 7% and 20% fewer targets than the control subjects. The study also calculated the efficiency of the reaching movement only for L–1, as it would not be possible to measure it when targets or obstacles were moving. The difference in efficiency between the two groups was statistically significant (see Table 4), with the control subjects being 26% more efficient than the PD-OFF group. The target order is another feature extracted to indicate the subject's cognitive ability to perform higher-level planning. The median R^2^ value for the PD-OFF group was 0.705, while the control subjects were closer to the ideal order with a median R^2^ value of 0.838 and the difference was statistically significant (see Table 4) between the two groups. The PD-OFF group exhibited 49% more endpoint error than the control group in trials where the targets were stationary, while the difference in error between PD-OFF and control groups was 10% when the targets were moving. The control subjects have statistically significant (see Table 4) lower endpoint error than the PD-OFF group in all four levels. The PD-OFF group hit more obstacles than the control subjects in all levels. The percentage difference in obstacle hits between PD-OFF and control groups across all four levels was 44%. The difference was statistically significant (see Table 4) in trials where the obstacles were moving. The relationship between the subject's performance and task complexity was investigated by calculating the slope between performance and Index of Difficulty (ID) based on Fitts's law^40,41^. The average rise in slope across all levels for the PD-OFF group when compared to the control group was 20%, indicating a steeper deterioration in performance among the PD-OFF group as task complexity increased.

The study also compared the performance of the participants from the perspective of the computational models. A comparison based on the minimum energy and variance model shows that the control subjects have the least endpoint variance across all levels, indicating that they can better plan and execute their movement than the PD-OFF group. The control subjects also exhibited 32% fewer speed peaks than the PD-OFF group, thereby closely following the minimum jerk model. Tables 5 and 6 show the correlation values used to compare the subject's performance from the perspective of the obstacle avoidance and minimum intervention model, respectively. When investigating the subject's ability to follow the obstacle avoidance model (see Table 5), the control subjects and PD-OFF group exhibited a positive correlation in L-4 and L-2, 4, respectively, thereby letting both parts of the cost function increase in these levels. While both PD-OFF and control groups could not reduce the cost function in L-4 where both target and obstacle were moving, only the PD-OFF group failed to reduce at least one part of the cost function in a slightly easier L-2 where only the target was moving. Moving to speed-to-accuracy trade-offs, the ratio of increase in error for every cm/s increase in mean speed (error-speed ratio), as shown in Table 4, was almost two times higher in the PD-OFF group compared to the control group. Compared from the perspective of the minimum intervention model using Table 6, the corrective movements performed by the PD-OFF group has a statistically significant positive correlation with obstacle hits indicating that the corrective movements did not help avoid obstacles. On the contrary, as they performed more corrective movements, they also hit more obstacles. However, the corrective movements performed by control subjects have a negative correlation with obstacle hits showing that as they performed more corrective movements, they hit fewer obstacles.

**Table 5:** Correlation between the endpoint variance and obstacle hit-to-warn ratio.

Group	L-1	L-2	L-3	L-4
PD-OFF	− 0.4497 (*p* = 0.0076*)	0.3764 (*p* = 0.0337*)	− 0.2421 (*p* = 0.0394*)	0.2153 (*p* = 0.0320*)
PD-ON	0.0966 (*p* = 0.6536)	0.2501 (*p* = 0.0941)	0.3091 (*p* = 0.0416*)	0.0853 (*p* = 0.6920)
Control	− 0.2143 (*p* = 0.0191*)	− 0.2857 (*p* = 0.0408*)	− 0.6429 (*p* = 0.0462*)	0.4286 (*p* = 0.0299*)

Spearman correlation was applied; the table indicates the correlation coefficient (${r}_{s}$) and the corresponding *p*-value; * indicates statistical significance.

**Table 6:** Correlation between corrective movement and target hit, corrective movement and obstacle hit.

Group	Correlation between corrective movements and target reach	Correlation between corrective movements and obstacle hit
PD-OFF	− 0.6328 (*p* = 0.0003*)	0.2771 (*p* = 0. 024*)
PD-ON	− 0.6540 (*p* = 0.0039*)	− 0.0445 (*p* = 0.271)
Control	− 0.1183 (*p* = 0.0848)	− 0.3618 (*p* = 0.031*)

Spearman correlation was applied; the table indicates the correlation coefficient (${r}_{s}$) and the corresponding *p*-value; * indicates statistical significance.

### PD-OFF vs. PD-ON subjects

The study investigated the effect of medication on the SMC deficits experienced due to PD. The medication mitigated the motor deficits experienced in PD as features such as mean speed, movement time, reaction time, and time to reach maximum speed improved in the PD-ON group across all levels by 10%, 6.65%, 16%, and 5.6%, respectively, compared to PD-OFF group.

The features investigating the sensory impairments worsened after medication in certain levels compared to the PD-OFF group. The obstacle hit-to-warn ratio was lower after medication when the obstacles were stationary (L-1 and L-2). However, the PD-ON group performed worse than the PD-OFF group when the obstacles were moving, as the obstacle hit-to-warn ratio increased by 12% and 7% in L-3 and L-4, respectively. The corrective time for perturbation was higher in the PD-ON group compared to the PD-OFF group in L-3 and L-4.

Looking at the effect of medication on a subject's cognitive ability, features such as target order and efficiency improved in the PD-ON group, although they were not statistically significant (see Table 4). The PD-ON group reached more targets than the PD-OFF group, as the target reach percentage increased by 10% for the PD-ON group across all levels, and the difference was statistically significant (see Table 4) in L-2 and L-4. However, the PD-ON group performed worse on certain features in levels where the cognitive load was much higher. The endpoint error for the PD-ON group worsened by 7.5% in L-2 compared to the PD-OFF group. Further, the PD-ON group also hit considerably more obstacles than the PD-OFF group in L-4. Interestingly, the PD-ON group exhibited higher error and obstacle hits than the PD-OFF group only in complex levels where obstacles or targets were moving and not in simpler levels.

We compared the results for PD-OFF and PD-ON groups from the perspective of six computational models. Looking at the results from the perspective of the minimum energy and variance model, the PD-ON group performed worse than the PD-OFF group in L-2 and L-4, as indicated by a statistically significant increase (see Table 4) in endpoint variance. Moving to the minimum jerk model, the PD-ON group has lesser speed peaks than the PD-OFF group in L-1, 2, and 3. In terms of the obstacle avoidance model, Table 5 shows that while both PD-OFF and PD-ON groups performed worse in L-2, only the PD-ON group also performed worse in L-3 in that both parts of the cost function increased, as indicated by the positive correlation. The error-speed ratio (see Table 4) calculated for the speed-accuracy trade-off model indicates an improvement of 9% across all levels after medication. In terms of the minimum intervention principle, while the PD-OFF group has a positive correlation (see Table 6), the PD-ON group has a negative correlation between the corrective movements and obstacle hits. Therefore, after medication, the patients improved as their corrective movements helped avoid obstacles, although this correlation was not statistically significant.

### PD-ON vs. control subjects

Compared with the PD-ON group, the performance of control subjects was much better across all the features and in all levels of the task. Looking at the motor deficits, features such as mean speed, movement time, reaction time, and time to reach maximum speed are 22%, 9.5%, 2.9%, and 11.4%, respectively, better in the control group than in the PD-ON group.

Features such as obstacle hit-to-warn ratio and corrective time for perturbation used to evaluate the sensory deficits were again 77% and 18%, respectively, better in the control group than the PD-ON group.

The cognitive features also show a substantial deterioration in the PD-ON group compared to the control group. Again, a trend of performance deterioration was seen as the task complexity increased when performing a within-group analysis for both groups. However, compared to the control group, the PD-ON group had a steeper deterioration in performance as the task complexity increased, which was indicated by an increase of 16% in the slope between performance and ID. Therefore, by comparing PD-ON and control groups, it was evident that the performance of PD patients with medication still did not match the performance of control subjects.

From the perspective of minimum variance and energy models, the endpoint variance for the PD-ON group was two times higher than the control group in L-1 and L-3, while the PD-ON group showed a 42% and 68% increase in endpoint variance for L-2 and L-4, respectively compared to control group. The speed peaks were also marginally higher in the PD-ON group compared to the control group when investigating the minimum jerk model. Looking at the correlation for the obstacle avoidance model (see Table 5), the PD-ON group performed worse than the control group in L-2 and L-3 as both parts of cost functions increased, while for the control group, at least one part of the cost function was reduced. Compared from the perspective of the speed-to-accuracy trade-off model, the error-speed ratio across all levels (see Table 4) was 53% higher in the PD-ON group compared to the control group. An analysis based on the minimum intervention principle, shown in Table 6, clearly shows a negative correlation between obstacle hit and corrective movements, indicating that the control group was able to effectively distinguish between task-relevant and irrelevant errors. While the correlation between obstacle hit and corrective movement for the PD-ON group was negative, it was not statistically significant.

### Correlation analysis

The correlation between the extracted features and the motor part of the UPDRS score in both ON and OFF states was calculated, and the correlation value is shown in the Supplementary material under Table S1. Of the 18 features, 11 and 6 features extracted during the OFF and ON states, respectively, have a statistically significant correlation (see Table S1) with the corresponding UPDRS-III score. Furthermore, the correlation between the MoCA score and the extracted features was also calculated and shown in the Supplementary material (see Table S2).

## Discussion

In this study, patients diagnosed with PD and healthy subjects were recruited to objectively examine deficits in movement planning and online error correction of upper-limb motion using a robotic manipulandum. The kinematic data collected from the robotic manipulandum was used to extract 18 features that act as performance indicators for motor, sensory and cognitive functions in planning and correcting upper-limb movements. A direct comparison between the PD and control groups indicated a deterioration in performance among the PD patients across multiple features. The effect of medication on these deficits was investigated. A computational model-based analysis was also performed to understand why such a deterioration in performance was seen in the PD group and how they differ from the control group in planning and correcting a movement. A correlation analysis was also done, which validated the method's ability to objectively characterize and individualize the deficits in PD patients^42^.

Numerous studies have discussed motor and cognitive dysfunctions in PD. However, various limitations in earlier studies needed to be addressed. Schneider et al.^14^ discussed the relationship between cognitive dysfunctions and motor symptoms. The limitation is that the study neither uses any objective measures nor explores any sensory and motor dysfunctions leading to SMC deficits. Another study^43^ by Desmurget et al. showed a lack of ability in PD patients to correct their movements when a target jump was induced. However, the target jump was always induced at the movement onset and therefore was not unexpected. The study also does not discuss the movement planning aspect of SMC. Gentilucci et al.^44^ have discussed the deficit among PD patients in retrieving the already generated motor plan. However, it does not investigate the deficit in generating or correcting the motor plan. Studies by Gaprielian et al.^15^ and Wiratman et al.^45^ analyzed features relating to motor symptoms such as mean speed, movement area, target, and obstacle hit. However, the studies only focused on well-known motor symptoms and did not analyze any complex features that could help study SMC deficits. Moreover, the obstacle avoidance task used in the studies^15,45^ has several limitations. The patients were tested only under a single condition with the obstacle and targets released from 10 static locations. In contrast, the task used in our study includes four different conditions to understand how the subjects adapt to a dynamic environment. Moreover, in earlier studies, no additional perturbation or warning signal was provided when nearing an obstacle which was vital in our study to investigate the subject's ability to perform corrective movements. No study has so far compared a subject's performance with any computational models to understand the basis of the deficits and the features that need to be targeted to improve movement planning and error correction in PD.

**Figure 4:**
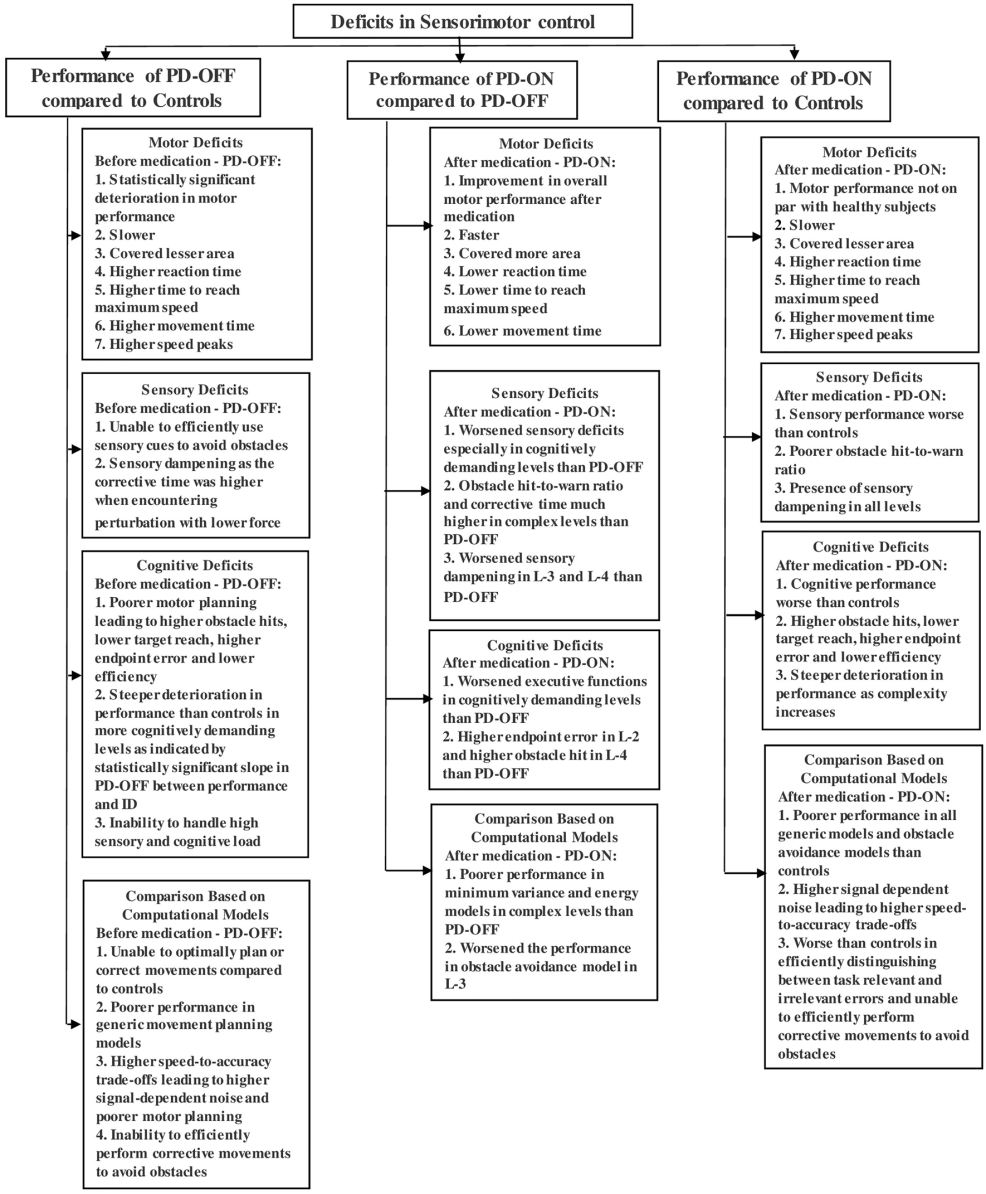
Summary of the study's findings.

Figure 4 summarizes the study's results. Compared with the control group, the PD-OFF group showed a deterioration in motor, cognitive and sensory performance resulting in an SMC deficit. The motor deficits in the PD-OFF group were very evident as they were much slower, had higher speed peaks, and covered less area on the screen. The study also detected impairments in appropriately interpreting or processing sensory information, as the PD-OFF group had a higher obstacle hit-to-warn ratio than the control group. The difference in the corrective time for perturbation between PD-OFF and control groups reduced as the force applied in a perturbation increased, indicating a sensory dampening due to PD. In other words, the PD-OFF group was much better at perceiving strong sensory information (high force) than a weak one, and therefore, their corrective time was much closer to the control group in levels where higher force was applied. Further, after experiencing the first perturbation, control subjects were more prepared and generated corrective movements much quicker than the PD-OFF group to handle further perturbations. A similar trend was observed in features evaluating cognitive deficits, where the control subjects outperformed the PD-OFF group in all the features. The findings indicated a deterioration in cognitive abilities, such as executive functions among PD-OFF, when performing complex motor tasks, as shown by the median scores in Table 4. This deterioration in executive functions was observed in a patient population with a mean and median MoCA score of 26. Furthermore, the correlation between the cognitive features and MoCA scores (Table S2) shows that certain cognitive features that were shown to be affected due to PD did not correlate with the MoCA scores. This may suggest that certain cognitive deficits that were not detected through subjective clinical assessments may be detected through an in-depth and objective analysis. As Koerts et al.^46^ have suggested, complementing the subjective assessments with an objective analysis may help in a better quantification of deficits as both assessments may provide valuable but different information about the deficits in PD patients. A common observation across multiple features was that there was a relationship between the subjects' performance with the cognitive and sensory load. That is, in complex trials that required the subjects to correct for multiple perturbations and tackle moving targets and obstacles, both groups performed much worse compared to their own performance in relatively simpler trials. However, the difference was that the PD-OFF group had a steeper deterioration in performance than the control group, as indicated by a higher slope between performance and ID. Therefore, it is evident that in addition to motor deficits, PD patients also experienced sensory and cognitive impairments and were also unable to efficiently handle high sensory and cognitive loads. A comparison between PD-OFF and control groups based on the computational models was also performed. The performance of the control group was substantially better than the PD-OFF group across all levels when compared from the perspective of generic models such as minimum variance, jerk, and energy models. In the obstacle avoidance model, the PD-OFF group performed worse than the control group in simpler trials from L-2, while both groups did not perform well in complex trials from L-4, indicating that the threshold of difficulty to abide by the computational model was much lower in PD patients than in control subjects. The PD-OFF group also exhibited poorer speed-to-accuracy trade-offs leading to higher signal-dependent noise, which could affect motor planning^29^. The comparison based on the minimum intervention principle showed a lack of ability among the PD-OFF group to perform corrective movements to avoid task-relevant errors as opposed to control subjects who were able to effectively avoid obstacles by performing corrective movements. To summarize, the PD patients did not entirely fail in abiding by the computational models. However, their ability to arrive at an optimal solution for movement planning and error correction seems to have diminished substantially compared to the control group.

A comparison between PD-ON and PD-OFF groups indicates that the medication has improved the patient's motor performance. However, it was accompanied by a worsening of sensory and cognitive functions, which are vital in planning and correcting complex task-specific movements. This was evident from the fact that the PD-ON group had a higher obstacle hit, endpoint error, and obstacle hit-to-warn ratio than the PD-OFF group in complex levels such as L-2, L-3, and L-4, which are cognitively more demanding than L-1 and required the participants to adapt to a dynamic environment and rely more on the sensory cues. Kulisevsky et al.^47^ have also discussed that PD patients after medication may perform differently in tasks requiring greater resources (more cognitively demanding) as opposed to tasks requiring fewer resources which aligns with our results. Thus, the medication negatively affected the patient's performance in trials that were more cognitively demanding. The medication also worsened the sensory dampening in patients as their corrective time for perturbation was much higher than their OFF state in L-3 and L-4. A few studies^48^ have also reported worsening of sensory impairments after medication, while some^49,50^ have reported no improvements in neuropsychological and cognitive performance after the medication. As shown in Fig. 4, the PD-ON performed worse than PD-OFF from the perspective of minimum energy and variance models in more cognitively demanding levels (L-2, L-4: levels where targets were moving), which matches our earlier observation. Again, in terms of obstacle avoidance models, the PD-ON group did worse than the PD-OFF group. After medication, both endpoint variance and obstacle hit-to-warn ratio increased in L-3, whereas one of these parameters in L-3 was reduced before medication. There were improvements observed in the error-speed ratio after medication. Therefore, there was performance deterioration observed after medication in most models relating to movement planning. However, there was a statistically insignificant improvement observed after medication in correcting task-relevant errors based on the minimum intervention model.

A comparison between PD-ON and control groups showed that while the medication improved motor features compared to the PD-OFF group, the performance of motor, sensory and cognitive systems in the PD-ON group was still less compared to those in a healthy subject. As shown in Fig. 4, the analysis comparing the performance of the PD-ON and control groups based on minimum energy and variance models shows that their endpoint variance was higher than the control group in all levels. With respect to the obstacle avoidance model, the PD-ON group did worse in L-2 and L-3 compared to the control subjects. The PD-ON group also exhibited a higher speed-to-accuracy trade-off than the control group across all levels. While the PD-ON group showed some improvement from the perspective of the minimum intervention principle, their correlation value was not statistically significant, indicating that the control subjects were still the most efficient in distinguishing between task-relevant and irrelevant errors.

Further, the study correlates the extracted features with the UPDRS—III, which has improved considerably after medication. The features that indicate motor performance correlated with UPDRS—III during the OFF state, while many features that indicate sensory and cognitive performance had no significant correlation. This shows that the progression of cognitive and sensory deficits may differ from that of the motor deficits, which aligns with earlier hypotheses^51,52^.

Summarizing all the results, in addition to motor deficits, the PD-OFF group also exhibited sensory impairments such as sensory dampening, deterioration of the cognitive ability to perform the goal-directed motion, and an inability to efficiently perform cognitively demanding tasks. Further, the PD-OFF group also struggled to optimally plan or correct movements from the perspective of the computational models compared to the control group, which led to their underperformance. Moving to the PD-ON group, the patients after medication exhibited better motor performance than the PD-OFF group. However, the medication worsened their sensory and cognitive impairments, and thereby the patients performed much worse than in the OFF state in more cognitively demanding levels. Further, the PD-ON group performed worse in certain features extracted based on movement planning models compared to the PD-OFF group. This may imply that while there is an important and positive motor performance impact, there may be an actual adverse effect in terms of task performance with the use of medications. This important observation needs to be considered during patient assessment and medication optimization. A comparison between the PD-ON group and the control group shows that the medication does not improve the patient's performance to the level of a healthy subject for any feature. Therefore, the study provides new insights into the deficits associated with executive and sensory functions when performing goal-directed movements and has also shown the effects of medication on SMC functions. Furthermore, the study also provides new insights into the deficits associated with cognitive abilities, such as executive functions, and how they affect movement expression. The need to understand this relationship has been indicated in a review by Ferrazzoli et al.^53^, as this understanding might be necessary to design tailored rehabilitation protocols.

Our study has a few limitations that will be addressed in future work. In this study, the features were categorized to evaluate the motor, sensory and cognitive deficits. While the extracted features were categorized to evaluate the motor, sensory and cognitive deficits based on earlier literature, and because these features are primarily influenced by the domain (motor, sensory or cognitive) that it was assigned to assess, it should also be noted that these features may also be influenced by deficits by other domains. For instance, obstacle hit, which was a cognitive feature, may have also been minorly influenced by motor deficits. This is one of the limitations of the study, and future work should focus on developing features that can be considered a pure measure of motor, sensory or cognitive functions. Therefore, further work is needed before using these features in a clinical setting for the diagnosis or management of PD. The extracted features pertaining to the right and left arm were averaged together when examining the SMC impairments in PD. Individual analysis of each arm (right and left) was not done as the handedness of the participants varied from one person to another. Therefore, separately examining the right and left arm would not take into account the performance difference between the dominant and non-dominant hand. However, future work necessitating a performance analysis of each arm separately would be needed to account for the diversity in the handedness of the participants. The study investigated only the upper-limb movements and did not investigate any lower-limb deficits in PD disease. Further, while the study provides sensory cues to help avoid obstacles, it did not explore which type of sensory cues (visual, haptic, or auditory) are more effective in PD. Understanding the type could help provide the appropriate sensory cues for PD patients during rehabilitation regimes. It should be noted that the robotic device used in this study only allows movements in two dimensions. While the results from this study can be extended to a three-dimensional space, a similar in-depth analysis in a three-dimensional environment might be needed to better understand the performance of PD patients in a more complex environment.

## Conclusion

This study used a robotic manipulandum for an objective investigation of SMC deficits focusing on movement planning and error correction of upper-limb motions in PD. The study addressed the following issues: (1) Characterize the motor, sensory and cognitive deficits that affect SMC functions such as movement planning and error correction in PD; (2) Effect of dopaminergic medication on these deficits; (3) Effect of PD from the perspective of the computational models, and comparison with healthy subjects. The results indicate a statistically significant deterioration of motor, sensory and cognitive performance in PD patients compared to the control group. Improvements were seen in the simple motor tasks with medications (PD-ON group). However, the treated patients performed worse when the tasks became complex and required engagement of the sensory-cognitive networks. This highlights the fact that the dysfunction in PD is not just motor but, in the SMC, and that the task performance may be significantly impaired due to the negative medication effects, despite UPDRS -III improvement. Comprehensive and multi-modal effects of PD and the equally complex effects of the medication will need to be considered to optimize the many treatments now available for PD. The insights provided through the objective assessment of SMC deficits and the analysis based on computational models may serve as a starting point in opening new doors for a more individualized and targeted treatment approach, thereby improving the overall efficacy of the treatment. The relation between cognitive deficits and movement expression has also been explored, i.e., how cognitive deficits affect the overall task performance, and understanding this motor-cognitive relationship may be vital in designing targeted treatments^53^. Furthermore, while there exists a limitation in the classification of features to assess motor, sensory and cognitive domains, the study does provide new insights into individually evaluating multiple domains associated with SMC functions using objective methodologies as certain deficits reported through in-depth, objective analysis may not be detected through subjective assessments. However, further work in this direction is needed before these metrics can be used for objective assessments in clinical practice.

## Supplementary Information

Supplementary Video 1.

Supplementary Information 1.

## Data Availability

The analyzed data that support the findings of this study are provided in the manuscript and the supplementary material. The raw kinematic data collected from the KINARM endpoint robot may be available from the corresponding author (ykrishn4@uwo.ca) upon reasonable request.

## Code availability

The Simulink model designed to construct the obstacle avoidance task may be made available from the corresponding author (ykrishn4@uwo.ca) upon reasonable request.
