# Supplementary material for: Robot-assisted investigation of sensorimotor control in Parkinson's disease
Source: Sci Rep. 2023 Mar 23;13:4751. doi: 10.1038/s41598-023-31299-z (PMC10036530; doi:10.1038/s41598-023-31299-z)
Supplement: Supplementary file 2 — Supplementary Information 1. [file 41598_2023_31299_MOESM2_ESM.pdf]

# Supplementary Material

# Robot-Assisted Investigation of Sensorimotor Control in Parkinson's Disease

Yokhesh K. Tamilselvam[1, *], Mandar Jog[2], and Rajni V. Patel[3]

[1*] Canadian Surgical Technologies and Advanced Robotics (CSTAR) and Department of Electrical and Computer Engineering, University of Western Ontario (UWO), London, Ontario N6A 5B9, Canada

[2] Department of Clinical Neurological Sciences, Department of Electrical and Computer Engineering, UWO, and the London Movement Disorders Centre, London, Ontario, Canada

[3] CSTAR, Department of Electrical and Computer Engineering, Department of Clinical Neurological Sciences, and Department of Surgery, UWO, London, Ontario, Canada

[*]ykrishn4@uwo.ca

| Features | Correlation with UPDRS score in OFF state ($r_s$) | Correlation with UPDRS score in ON state ($r_s$) |
|---|---|---|
| Mean speed | -0.4749 ($p$ = 0.010) | -0.2322 ($p$ = 0.042) |
| Peak speed | -0.3967 ($p$ = 0.014) | -0.2295 ($p$ = 0.046) |
| Time to reach maximum speed | 0.3231 ($p$ = 0.021) | 0.3145 ($p$ = 0.019) |
| Movement area | -0.3665 ($p$ = 0.013) | -0.2310 ($p$ = 0.042) |
| Reaction time | 0.2926 ($p$ = 0.030) | NS |
| Speed peaks | 0.6417 ($p$ < 0.001) | 0.5124 ($p$ < 0.001) |
| Movement time | 0.3114 ($p$ = 0.023) | 0.4514 ($p$ < 0.001) |
| Obstacle hit to warn ratio | NS | NS |
| Corrective time for perturbation | NS | NS |
| Target reach percent | -0.5717 ($p$ = 0.005) | NS |
| Efficiency | -0.5253 ($p$ = 0.008) | NS |
| Target order | NS | NS |
| Endpoint error | 0.2975 ($p$ = 0.034) | NS |
| Obstacle hit | NS | NS |
| Corrective movements | NS | NS |
| Endpoint variance | NS | NS |
| Slope between performance and index of difficulty | NS | NS |
| Error-speed Ratio | 0.3528 ($p$ = 0.019) | NS |

Note: Spearman Correlation was applied; $p$-values less than 0.001 are indicated as $p$<0.001

**Table S1**: Correlation between UPDRS and Extracted Features

| Features | Correlation with MoCA score in OFF state ($r_s$) | Correlation with MoCA score in ON state ($r_s$) |
|---|---|---|
| Mean speed | NS | NS |
| Peak speed | NS | NS |
| Time to reach maximum speed | NS | NS |
| Movement area | NS | NS |
| Reaction time | -0.3085 ($p$ = 0.026) | -0.3146 ($p$ = 0.023) |
| Speed peaks | NS | NS |
| Movement time | NS | NS |
| Obstacle hit to warn ratio | NS | NS |
| Corrective time for perturbation | NS | NS |
| Target reach percent | 0.3571 ($p$ = 0.021) | 0.2988 ($p$ = 0.026) |
| Efficiency | NS | 0.3123 ($p$ = 0.020) |
| Target order | NS | NS |
| Endpoint error | -0.2374 ($p$ = 0.044) | -0.3908 ($p$ = 0.003) |
| Obstacle hit | NS | NS |
| Corrective movements | NS | NS |
| Endpoint variance | -0.3339 ($p$ = 0.020) | -0.4785 ($p$ < 0.001) |
| Slope between performance and index of difficulty | NS | NS |
| Error-speed Ratio | NS | NS |

Note: Spearman Correlation was applied; $p$-values less than 0.001 are indicated as $p$<0.001

**Table S2**: Correlation between MoCA and Extracted Features

# Method used to calculate the Index of Difficulty (ID)

Equation S1 was used to calculate the ID that was used to obtain the slope between the performance indicator (movement time) and the ID.

$$ID = ax^2 + bx + c \quad \text{(S1)}$$

$$x = log\left(\frac{2A}{w}\right) + T * exp\left(\frac{s}{100}\right) \quad \text{(S2)}$$

where $A$ is the movement amplitude, $w$ is the target width, and $s$ is the speed of the objects, $T$ is 1 if the objects are moving, and $T$ is 0 if the objects are stationary.

| Patient number | Gender | Age at recruitment (years) | Years with disease (years) | MoCA | UPDRS-III (OFF-L-dopa) | UPDRS-III (ON-L-dopa) |
|---|---|---|---|---|---|---|
| PD-01 | F | 55 | 3 | 29 | 43 | 18 |
| PD-02 | M | 49 | 5 | 29 | 9 | 4 |
| PD-03 | M | 60 | 7 | 29 | 28 | 16 |
| PD-04 | M | 78 | 8 | 30 | 42 | 27 |
| PD-05 | F | 60 | 12 | 28 | 36 | 5 |
| PD-06 | M | 59 | 11 | 29 | 63 | 42 |
| PD-07 | M | 78 | 7 | 30 | 34 | 20 |
| PD-08 | M | 53 | 2 | 28 | 39 | 16 |
| PD-09 | F | 60 | 6 | 28 | 26 | 8 |
| PD-10 | M | 77 | 3 | 27 | 61 | 51 |
| PD-11 | F | 60 | 8 | 25 | 31 | 27 |
| PD-12 | M | 77 | 30 | 22 | 76 | 51 |
| PD-13 | M | 67 | 8 | 28 | 24 | 19 |
| PD-14 | M | 57 | 6 | 26 | 68 | 51 |
| PD-15 | M | 71 | 7 | 30 | 36 | 29 |
| PD-16 | M | 74 | 10 | 21 | 49 | 38.5 |
| PD-17 | M | 60 | 9 | 26 | 45 | 38 |
| PD-18 | M | 78 | 8 | 24 | 39 | 30.5 |
| PD-19 | M | 76 | 8 | 30 | 41 | 36 |
| PD-20 | M | 65 | 11 | 28 | 42 | 30 |
| PD-21 | M | 49 | 8 | 28 | 56 | 26 |
| PD-22 | M | 70 | 17 | 26 | 39 | 17.5 |
| PD-23 | M | 55 | 6 | 26 | 48 | 40 |
| PD-24 | M | 74 | 12 | 25 | 43 | 33 |
| PD-25 | M | 61 | 7 | 29 | 42 | 25 |
| PD-26 | M | 73 | 16 | 30 | 44 | 24 |
| PD-27 | F | 49 | 10 | 28 | 36 | 31 |
| PD-28 | M | 54 | 9 | 26 | 38 | 22 |
| PD-29 | M | 49 | 8 | 24 | 59 | 33 |
| PD-30 | M | 66 | 8 | 26 | 58 | 36 |
| PD-31 | M | 59 | 8 | 21 | 50 | 33 |
| PD-32 | F | 73 | 9 | 25 | 42 | 26 |
| PD-33 | F | 71 | 18 | 29 | 46 | 27 |
| PD-34 | M | 67 | 10 | 25 | 41 | 26 |
| PD-35 | M | 61 | 9 | 28 | 54 | 48 |
| PD-36 | F | 51 | 4 | 28 | 35 | 19 |
| PD-37 | F | 49 | 3 | 26 | 56 | 38 |
| PD-38 | M | 69 | 9 | 28 | 52 | 38 |
| PD-39 | M | 62 | 11 | 26 | 62 | 44 |
| PD-40 | M | 62 | 11 | 21 | 49 | 33 |
| PD-41 | F | 54 | 8 | 30 | 35 | 24 |

| | | | | | | |
|---|---|---|---|---|---|---|
| PD-42 | F | 61 | 9 | 29 | 38 | 28 |
| PD-43 | F | 61 | 11 | 21 | 59 | 42 |
| PD-44 | F | 62 | 12 | 24 | 54 | 25 |
| PD-45 | M | 72 | 11 | 21 | 52 | 43 |
| PD-46 | F | 57 | 8 | 30 | 31 | 9 |
| PD-47 | M | 63 | 13 | 24 | 49 | 26 |
| PD-48 | M | 58 | 8 | 21 | 44 | 27 |
| PD-49 | F | 61 | 9 | 21 | 47 | 30 |
| PD-50 | M | 62 | 10 | 24 | 49 | 38 |
| PD-51 | M | 67 | 8 | 24 | 53 | 36 |
| PD-52 | M | 65 | 8 | 26 | 44 | 28 |
| PD-53 | M | 63 | 9 | 26 | 62 | 31 |
| PD-54 | M | 71 | 14 | 29 | 42 | 30 |
| PD-55 | F | 53 | 8 | 22 | 49 | 28 |
| PD-56 | M | 57 | 2 | 27 | 41 | 30 |

**Table S3**: Clinical and Demographic information for the PD subjects

# Supplementary Video

The supplementary material also includes a video showing the design of each level of the custom-built Obstacle avoidance task used in this study.

**Video Legend or title**: Design of the custom-built Obstacle Avoidance Task
